# Atrial fibrillation inpatient management patterns and clinical outcomes during the conflict in Syria: An observational cohort study

**DOI:** 10.1177/02676591241259140

**Published:** 2024-06-03

**Authors:** Ibrahim Antoun, Majed Aljabal, Alkassem Alkhayer, Yaman Mahfoud, Alamer Alkhayer, Peter Simon, Ahmed Kotb, Joseph Barker, Akash Mavilakandy, Muhammad Usman Naseer, Riyaz Somani, G André Ng, Mustafa Zakkar

**Affiliations:** 1 Department of Cardiovascular Sciences, 150459 University of Leicester, Leicester, UK; 2 Faculty of Medicine, University of Aleppo, Aleppo, Syria; 3 9632 Department of Psychiatry, Leicestershire Partnership NHS Trust, Leicester, UK; 4 594809 Department of Medicine, University of Tishreen’s Hospital, Latakia, Syria; 5 Department of Research, National Heart and Lung Institute, 90897 Imperial College London, London, UK; 6 Department of Cardiology, University Hospitals of Leicester NHS Trust, 156757 Glenfield Hospital, Leicester, UK; 7 NIHR Leicester Biomedical Research Centre, Leicester, UK; 8 Department of Cardiac Surgery, University Hospitals of Leicester NHS Trust, 156757 Glenfield Hospital, Leicester, UK; 9 Faculty of Medicine, University of Damascus, Damascus, Syria

**Keywords:** Syria, atrial fibrillation, rhythm control

## Abstract

**Background:**

Atrial fibrillation (AF) is the most common sustained arrhythmia worldwide. However, there is no data on AF inpatient management strategies and clinical outcomes in Syria.

**Objectives:**

The study aims were to review the inpatient management of patients with AF and assess cardiovascular (CV) mortality in a tertiary cardiology centre in Latakia, Syria.

**Methods:**

A single-centre retrospective observational cohort study was conducted at Tishreen’s University Hospital, Latakia, Syria, from June 2021 to June 2023. Patients ≥16 years of age presenting and being treated for AF as the primary diagnosis with or without a thromboembolic event were included. Medical records were examined for patients’ demographics, laboratory results, treatment plans and inpatient details. Studied outcomes include inpatient all-cause and CV mortality, ischemic and bleeding events, and conversion to sinus rhythm (SR).

**Results:**

The study included 596 patients. The median age was 58, and 61% were males. 121 patients (20.3%) were known to have AF. A rhythm control strategy was pursued in 39% of patients. Ischemic and bleeding events occurred in 62 (11%) and 12 (2%), respectively. CV and all-cause mortality occurred in 28 (4.7%) and 31 patients (5%), respectively. The presence of valvular heart disease (VHD) (adjusted odds ratio (aOR) = 9.1, 95% confidence interval (CI): 1.7 to 55.1, *p* < .001), thyroid disease (aOR: 9.7, 95% CI = 1.2 to 91.6, *p* < .001) and chronic obstructive pulmonary disease (COPD) (aOR: 82, 95% CI: 12.7 to 71, *p* < .001) were independent risk factors of increased CV inpatient mortality.

**Conclusion:**

Syrian inpatients admitted with AF in Latakia are relatively younger than those in other countries. Active thyroid disease, COPD and VHD were independent risk factors of inpatient CV mortality with AF.

## Highlights

• Inpatient cardiovascular mortality in this cohort was 4.7%.
• Smoking, thyroid disease, chronic lung disease and valvular heart disease were independent risk factors of inpatient cardiovascular mortality in patients presenting acutely with atrial fibrillation.
• Thromboembolic event incidence was high compared to the corresponding CHA_2_DS_2_-VASc score.
• The elective rhythm control strategy was challenging due to limited resources.

## Introduction

Atrial fibrillation (AF) is the most common sustained arrhythmia worldwide, and its prevalence in low to middle-income countries is likely underestimated.^1^ Although AF in the developed world is extensively studied, there is little data on AF demographics and management in the Middle East, with only four epidemiological data registries.^2^ AF-related research in the Arab world contributed only 0.7% of the total AF research.^3^

Syria has been embroiled in conflict since 2011. It has been deprived of healthcare resources and funding, particularly exacerbated during the Coronavirus disease 2019 (COVID-19) pandemic. As a result, less than half of its hospitals operate at usual performance, with over 50% of its healthcare workforce forced to leave due to conflict.^4^ More than half of the Syrian population lives in poverty,^5^ which is suggested to increase AF risk.^6^ AF management in hospitals during the current political and economic turmoil is unclear, with a lack of published inpatient outcomes and figures originating from Syrian hospitals. In the context of the resource constraints, a real-world depiction of the current practice of AF care and observed outcomes could potentially aid the management and allocation of resources by identifying remediable areas of deficiencies and, more importantly, reasonable and practical solutions that could be implemented.

This study aims to describe the characteristics and outcomes of patients treated for AF.

## Methods

This is a single-centre retrospective observational cohort study conducted at Tishreen’s University Hospital, Latakia, Syria, between the 1st of June 2021 and the 1st of June 2023. The project was conducted as a part of an audit approved by the hospital board (reference: 277/A).

Inclusion criteria were patients (over the age of 16 years) presenting to the hospital and treated for AF as the primary diagnosis or confirmed thromboembolic event thought to be due to confirmed AF by two different physicians. Patients exhibiting AF as a secondary component to a condition other than a thromboembolic event or complication to another primary diagnosis were excluded. The diagnosis of AF was established by 12-lead electrocardiograms (ECG), which were subsequently interpreted and verified by a consultant cardiologist.

Patient demographics, admission details, 12-lead ECGs, blood results, radiological reports and clinical outcomes during hospitalisation were extracted from patients’ records, recorded on a detected Excel sheet and stored securely. The outcomes studied were in-hospital cardiovascular (CV) mortality, all-cause mortality, inpatient conversion to sinus rhythm (SR) during admission, and inpatient ischemic and bleeding events. Ischemic events were defined by a transient ischemic attack (TIA) or a stroke based on clinical suspicion without the necessity of intracranial imaging or an extracranial thromboembolic event confirmed by imaging. A bleeding event was defined by any overt, actionable sign of haemorrhage with more bleeding than expected for a clinical circumstance, including bleeding found by imaging alone.

The study was written according to STROBE guidelines — see Supplemental Table 1.^7^

## Statistical analysis

Continuous variables are expressed as median and interquartile ranges (IQR). Categorical variables are expressed as counts and percentages (%). Pearson’s χ 2 or Fisher’s exact test was used for categorical variables between groups. Students’ t-tests and Kruskal-Wallis tests were used to compare continuous variables between the groups depending on the normality of the distribution.

Multiple logistic regression models were used to investigate the relationship between inpatient all-cause mortality, CV mortality, thromboembolic events, bleeding events and variables, with the results being presented as adjusted odds ratio (aOR) and 95% confidence interval (CI) were demonstrated. The multivariable models involved significant factors in the univariable analysis added to a base model of age and sex. We hypothesised that specific demographic characteristics and comorbidities would affect AF mortality and SR reversion. Therefore, a base model was constructed consisting of age and gender to assess the incremental value of comorbidities that are significantly associated with mortality. These comorbidities were then added to the base model to improve the mortality predictability. The new model’s cumulative discrimination was measured using the area under the curve; its statistical significance compared to the base model was assessed with the likelihood ratio test. A 2-sided *p*-value <.05 was considered statistically significant. Statistical analysis was performed using GraphPad Prism V10.0 for Mac (San Diego, California, USA).

## Results

### Baseline characteristics, presentation, and diagnostics tests

Between June 1st, 2021 and June 1st, 2023, 596 patients presented to the hospital with AF as the primary diagnosis on admission. Baseline characteristics are demonstrated in Table 1. All of whom were from an Arabic background. 121 patients (20%) were previously known to have AF on presentation. Paroxysmal and persistent AF were not differentiated due to the lack of Holter monitoring or previous clinic follow-up. The most common presenting complaint was palpitation in 302 patients (53%), followed by shortness of breath in 151 patients (27%), ischemic stroke and TIA diagnosed based on clinical suspicion in 20 (3%) and 22 patients (4%), respectively. Computed tomography (CT) scan of the head was only done in 12 patients (27%) due to the lack of resources, with half the scans showing evidence of an ischemic stroke and the rest were negative. Extracranial thromboembolic events occurred in 10 patients (2%). They all involved the lower limbs and were confirmed by a CT angiogram. Presenting complaints also included chest pain in 40 patients (7%), clinical heart failure in 28 patients (5%), and syncope in 24 patients (4%). Smoking was prevalent in 232 patients (74%), and moderate to severe valvular heart disease (VHD) was present in 90 patients (15%), of which 20 (22%) had rheumatic mitral stenosis, 18 (20%) had moderate to severe tricuspid regurgitation, 29 (21%) had moderate to severe mitral regurgitation, 30 (33%) had moderate to severe aortic stenosis, and three (3%) had surgically corrected metallic aortic valve. This information was extracted from previous echocardiograms that patients underwent. Only 3 out of 20 patients (15%) with active thyroid disease were on medical treatment. Demographic and lab results are demonstrated in Table 1. Potassium below 3.5 mmol/L was found in 98 patients (16%), while magnesium below 0.7 mmol/L was found in 77 patients (13%).

**Table 1.** Patient details, admission, vital signs and lab workup stratified by admission time.

	Total (*n* = 596)
Age (years)	60 (51-65)
Male	366 (61%)
Current smoking	232 (74%)
Hypertension	220 (37%)
Ischemic heart disease	101 (17%)
Diabetes mellitus	136 (23%)
Cerebrovascular disease	108 (18%)
Chronic obstructive lung disease	51 (9%)
Heart failure	108 (18%)
Thyroid disease	20 (3%)
CHA_2_DS_2_-VASc score	2 (1-3)
ORBIT-AF score	1 (0-2)
Valvular heart disease	90 (15%)
Haemoglobin (g/dL)	11 (9-13)
C-reactive protein (mg/dL)	40 (22-65)
White cell count (10^9^/L)	9 (6-12)
Rhythm control	230 (39%)
Magnesium (mmol/L)	0.9 (0.6-1.1)
Potassium (mmol/L)	4 (.4-4.6)

### Treatments

Management strategies were stratified into rhythm control, rate control or a combined approach based on clinical judgement and available resources. Both approaches included correcting potassium <3.5 mmol/L with an intravenous (IV) 40 mmol of potassium mixed with 1 L of 0.9% sodium chloride followed by a repeat blood test the next day with a repeat dose if potassium remains <3.5 mmol/L. Similarly, in patients with magnesium below 0.7 mmol/L, 4 mmol of magnesium sulphate 50% mixed with 250 mL of 0.9% sodium chloride was administered over 6 h with a repeat test the next day and a repeat dose if magnesium remained below 0.7 mmol/L. A rhythm control strategy was adopted in 230 patients (39%). This was done using pharmacological therapy, with amiodarone as the only available anti-arrhythmic agent. The regime used for all patients was IV loading dose of 300 mg followed by an oral maintenance dose of 200 mg twice a day (BD) for a week, then 200 mg once daily (OD) afterwards. Direct current cardioversion (DCCV) was attempted in an emergency setting for ten patients (2%) during admission due to hemodynamic instability, with eight (80%) being successfully cardioverted after a single shock and two patient deaths due to degeneration into asystole. Elective DCCV was not used in this cohort, nor were any patients scheduled for one after discharge. None of the patients were referred for catheter ablation or left atrial appendage closure, as it was unavailable locally. Monotherapy rate control was employed in 366 patients (61%). Metoprolol was the only IV rate-control drug available, and it was used in 90 patients (15%) with 5 mg to 15 mg boluses. The two other available oral drugs were bisoprolol 2.5-10 mg, used in 249 patients (42%), followed by digoxin (loading dose of 1 mg followed by 62.5-125 mcg daily), used in 28 patients (5%). Bisoprolol was used along with amiodarone for additional rate control in 131 patients (57% of amiodarone patients). Digoxin was not used with amiodarone in this cohort. The heart rate reduced significantly in all patients on discharge from 137 (120-152) beats per minute (BPM) to 76 (65-90) BPM, *p* < .0001.

Oral anticoagulation was used in 500 patients (84%) based on the CHA_2_DS_2_-VASc score (cut-offs of ≥1 for males and ≥2 for females as the threshold for anticoagulation)^8^ or if a rhythm control strategy was adopted.^9^ Of these, Warfarin was used in 21 patients (4%), of which 18 had rheumatic heart disease, and three had a metallic heart valve. The rest of the patients (*n* = 479) received direct oral anticoagulant (DOAC) based on availability. The available DOACs were apixaban (2.5-5 mg BD) and rivaroxaban (15-20 mg OD).

## Inpatient outcomes and predictors of outcomes

Outcomes are summarised in Table 2. Conversion to sinus rhythm (SR) was achieved during admission in 103 patients (18%) and was maintained until discharge, indicating a successful outcome. Of these patients, 58 had a rhythm control strategy (54%). Patients who converted to SR had a lower percentage of chronic obstructive pulmonary disease (COPD), VHD, and current smoking. Patients with SR conversion had more rhythm control than rate control (56% vs 35%, *p* < .001).

**Table 2.** Demographics and characteristics of patients stratified by conversion to sinus rhythm at any point during admission.

	Remained in AF (*n* = 493)	Converted to sinus rhythm (103)	*p*-value
Age (years)	60 (52-68)	60 (53-64)	0.378
Male	277 (35%)	89 (86%)	0.145
Current smoking	201 (41%)	31 (30%)	<0.001
Hypertension	180 (37%)	40 (39%)	0.672
Ischemic heart disease	88 (18%)	13 (13%)	0.442
Diabetes mellitus	120 (24%)	16 (16%)	0.536
Cerebrovascular disease	92 (19%)	16 (16%)	0.149
COPD	50 (1%)	1 (1%)	<0.001
Heart failure	92 (19%)	16 (16%)	0.171
Thyroid disease	18 (4%)	2 (2%)	0.559
CHA_2_DS_2_-VASc score	2 (1-3)	2 (1-3)	0.654
ORBIT score	2 (1-2)	1 (0-1)	0.886
Valvular heart disease	84 (17%)	6 (6%)	<0.001
Haemoglobin (g/dL)	11 (13-16)	12 (9-14)	0.416
C-reactive protein (mg/dL)	45 (40-54)	38 (35-41)	0.084
White cell count (10^9^/L)	11 (10-13)	8 (7-9)	0.501
Rhythm control	172 (35%)	58 (56%)	<0.001
Magnesium (mmol/L)	0.8 (0.6-1.1)	0.9 (0.8-1.1)	0.764
Potassium (mmol/L)	3.8 (3.4-4)	4.1 (3.9-4.2)	0.814

AF: atrial fibrillation. Chronic obstructive pulmonary disease.

In addition to the patients who were admitted with ischemic events, 12 had further thromboembolic events after 2 days^1–3^ of admission, including nine ischemic strokes, raising the total thromboembolic events in this cohort to 63 patients (11%).

Urological and gastrointestinal bleeds occurred in 12 patients (2%) within 36 h of anticoagulation initiation, but none were fatal. Eight patients resumed lower-dose DOAC after patient counselling and shared decision-making, while the rest discontinued, accepting the risk of cardioembolic events. In patients who had clinical ischemic strokes, DOAC was delayed 48 h after admission and was started along with clopidogrel lifelong. The median ORBIT-AF score^9^ was not significantly different between patients with bleeding events and those without (1 vs 2, *p* = .84).

**Figure 1.**
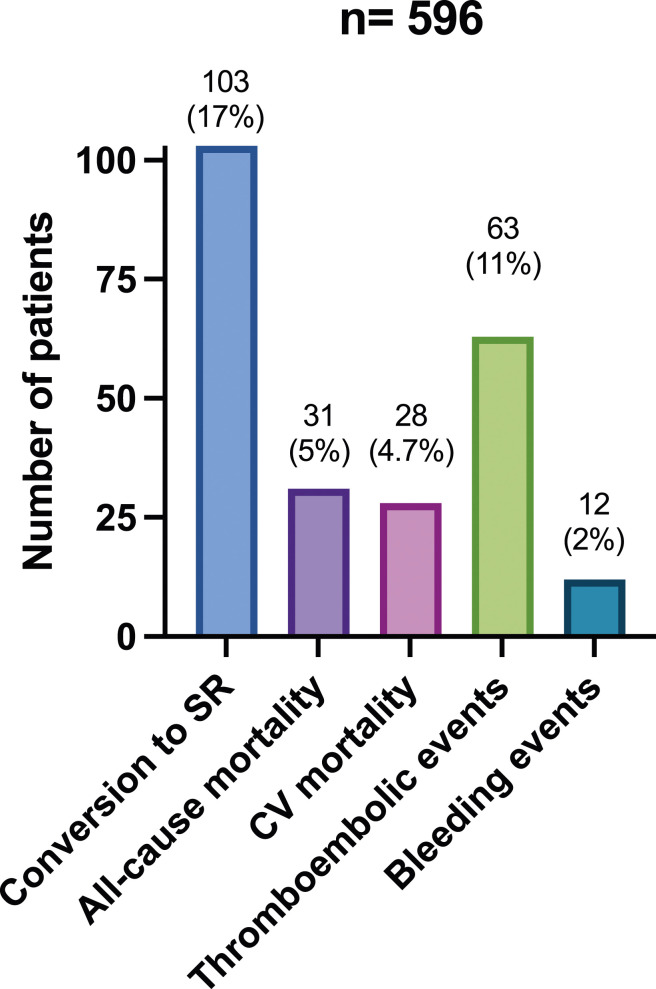
Summary of outcomes of interest for 596 consecutive patients admitted to Tishreen’s University Hospital and treated for atrial fibrillation between June 2021 and June 2023. SR: sinus rhythm. CV: cardiovascular.

Inpatient all-cause mortality was 5% (*n* = 31) after a median hospital admission of 4 days^3–7^. CV mortality occurred in 28 patients (4.7%), and the remaining three died from hospital-acquired infections (0.3%). None of the patients with CV mortality reverted to SR during admission; only three had a rhythm control strategy (11%). The remaining patients were discharged home after a median of 4^2–5^ days. Limited facilities and resources did not allow patients to be scheduled for an outpatient follow-up or diagnostic tests such as a Holter monitor or a transthoracic echocardiogram. A summary of outcomes of interest in this study is demonstrated in Figure 1. The first part of Table 3 demonstrates univariable and multivariable logistic regressions concerning all-cause mortality and CV mortality, while its second part shows univariable and multivariable logistic regressions concerning thromboembolic and bleeding events. The univariable logistic regression model showed that VHD, COPD, ischemic heart disease (IHD), VHD and thyroid disease were associated with increased inpatient all-cause mortality and CV mortality. When these factors were added to a multivariable model with a base model of age and sex, it demonstrated the presence of VHD (aOR = 9.1, 95% CI: 1.7 to 55.1, *p* < .001), thyroid disease (aOR: 9.7, 95% CI = 1.2 to 91.6, *p* < .001) and COPD (aOR: 82, 95% CI: 12.7 to 71, *p* < .001) were independent risk factors of increased CV inpatient mortality. Similarly, the presence of VHD (aOR = 8.5, 95% CI: 1.2 to 52.4, *p* < .001), thyroid disease (aOR = 9.1, 95% CI: 1.1 to 87.3, *p* < .001) and COPD (aOR = 77.2, 95% CI: 11.3 to 65, *p* < .001) were independently associated with an increased risk of all-cause mortality. A higher CHA2S2Vasc score was associated with increased thromboembolic events with an odds ratio of 2.3 and 95% CI 1.9 to 2.9. Multivariable analysis was not conducted because the CHA2S2Vasc score depends on age and sex (base model). No factors were associated with increased bleeding risk.

**Table 3.** The first part of the table shows univariable and multivariable logistic regressions concerning inpatient all-cause and cardiovascular mortality. The second part of the table demonstrates univariable and multivariable regression concerning bleeding and thromboembolic events. Variables that showed a statistically significant correlation with length of stay in the univariable analysis were included in the multivariable analysis, along with age and sex (base model).

	All cause-mortality				Cardiovascular mortality			
	Univariable analysis		Multivariable analysis		Univariable analysis		Multivariable analysis	
Variables	OR (95%CI)	*p*-value	aOR (95%CI)	*p*-value	OR (95%CI)	*p*-value	aOR (95%CI)	*p*-value
Age (per year increase)	1.2 (1-1.2)	0.062	1.1 (0.9-1.2)	0.09	1.1 (1-1.1)	0.066	1.1 (0.9-1.2)	0.09
Sex (male compared to female)	1.3 (0.6-3.2)	0.391	2.2 (0.3-16.6)	0.4	1.4 (0.62-3.1)	0.416	2.2 (0.3-16.6)	0.4
Hypertension (yes vs no)	1 (0.32-1.2)	0.732			0.9 (0.38-2)	0.801		
Diabetes mellitus (yes vs no)	0.72 (0.2-1.2)	0.179			0.76 (0.1-1.3)	0.172		
Cerebrovascular event (yes vs no)	0.72 (0.13-1.7)	0.372			0.77 (0.13-1.7)	0.375		
Ischemic heart disease (yes vs no)	3.1 (0.3-7.7)	0.045	1.9 (0.9-3.5)	0.092	3.3 (0.4-7.6)	0.047	2 (0.95-3.4)	0.083
Heart failure (yes vs no)	1.3 (0.49-3.5)	0.475			1.4 (0.54-3.3)	0.442		
Valvular heart disease (yes vs no)	13 (5.8-33)	<0.001	8.5 (1.2-52.4)	<0.001	13 (5.8-33)	<0.001	9.1 (1.7-55.1)	<0.001
Chronic obstructive lung disease (yes vs no)	96 (32-47)	<0.001	77.2 (11.3-65)	<0.001	98 (34-55)	<0.001	82.0 (12.7-71)	<0.001
CHA2S2Vasc score (per 1 point increase)	1.1 (0.76-1.6)	0.462			1.1 (0.88-1.5)	0.315		
Thyroid disease (yes vs no)	47 (14-78)	<0.001	9.1 (1.1-87.3)	<0.001	49 (15-76)	<0.001	9.7 (1.2-91.6)	<0.001
Smoking (yes vs no)	2.4 (0.9-6.6)	0.059			2.5 (1-6.9)	0.054		
White cell count (per unit-10^9^/l increase)	1.1 (0.98-1.2)	0.325			1.1 (0.95-1.2)	0.339		
Haemoglobin (per unit- g/dL increase)	1.2 (1.1-1.4)	0.100			1.2 (1-1.4)	0.092		
C-reactive protein (per unit- mg/dL increase)	1 (0.99-1)	0.424			1 (0.99-1)	0.344		
Calcium (per unit- mmol/L increase)	0.7 (0.07-12)	0.091			0.65 (0.06-13)	0.072		
Magnesium (per unit- mmol/L increase)	1.6 (0.5-5.8)	0.324			1.5 (0.4-6)	0.505		
Potassium (per unit- mmol/L increase)	1 (0.7-1.4)	0.923			0.95 (0.6-1.6)	0.847		
Heart rate (per beats per minute increase)	1 (0.97-1)	0.542			1 (0.99-1)	0.358		

Variables	Thromboembolic events		Bleeding events	
	Univariable analysis^a^		Univariable analysis^b^	
	OR (95% CI)	*p*-value	OR (95% CI)	*p*-value
Age (per year increase)	1.1 (0.8-1.2)	0.934	0.96 (0.92-1)	0.155
Sex (male compared to female)	1.1 (0.8-1.7)	0.764	0.52 (0.12-1.8)	0.338
Hypertension (yes vs no)	1 (0.32-1.2)	0.732	1.7 (0.45-5.6)	0.347
Diabetes mellitus (yes vs no)	1.5 (0.8-2.6)	0.200	0.3 (0.02-6)	0.180
Cerebrovascular event (yes vs no)	1.6 (0.6-2.2)	0.092	0.77 (0.13-1.7)	0.371
Ischemic heart disease (yes vs no)	1.2 (0.6-2-3)	0.598	0.98 (0.15-3.8)	0.980
Heart failure (yes vs no)	1.8 (1-3.3)	0.050	0.75 (0.11-2.9)	0.712
Valvular heart disease (yes vs no)	0.94 (0.42-1.9)	0.881	1 (0.9-1)	0.977
Chronic obstructive lung disease (yes vs no)	1.9 (0.9-4)	0.061	1 (0.95-1)	0.958
CHA2S2Vasc score (per 1 point increase)^a^	2.3 (1.9-2.9)	<0.001	0.68 (0.41-1)	0.105
ORBIT-AF score (per 1 point increase)	0.9 (0.7-1.3)	0.748	1.4 (0.7-3.2)	0.192
Thyroid disease (yes vs no)	0.93 (0.14-3.4)	0.923	1 (0.98-1)	0.998
Smoking (yes vs no)	1.1 (1-1.2)	0.954	1 (0.9-1)	0.978
White cell count (per unit-10^9^/l increase)	1 (0.99-1)	0.997	0.96 (0.8-1)	0.575
Haemoglobin (per unit- g/dL increase)	0.93 (0.86-1)	0.122	1.1 (0.99-1.5)	0.773
C-reactive protein (per unit- mg/dL increase)	1 (0.99-1)	0.105	1 (0.98-1)	0.618
Calcium (per unit- mmol/L increase)	0.58 (0.15-2.1)	0.410	1.3 (0.06-9)	0.560
Magnesium (per unit- mmol/L increase)	1 (0.41-2.6)	0.966	0.36 (0.4-2.6)	0.310
Potassium (per unit- mmol/L increase)	0.9 (0.71-1.6)	0.814	0.88 (0.37-2.1)	0.776

OR: odds ratio. aOR: adjusted odds ratio. CI: confidence interval.

^a^Multivariable analysis was not done for thromboembolic events due to the CHA2S2Vasc score being dependent on the base model of age and sex.

^b^Multivariable analysis was done for bleeding events due to the lack of significantly correlated variables in univariable analysis.

## Discussion

This is the first study to address AF inpatient treatment patterns and clinical outcomes in Syria, a country suffering from conflict since 2011. We report on an AF inpatient cohort of 596 patients. The main findings highlight that hospitalised Syrian patients with AF were relatively younger than those in neighbouring countries, with no elective rhythm control strategy employed, regardless of patient’s age, comorbidities or symptom burden due to the lack of resources available, with a rate control strategy adopted in most patients. Smoking, thyroid disease, COPD and VHD were independent risk factors of CV inpatient mortality.

There has been a significant increase in AF prevalence in low-income countries in the last 20 years.^10^ The Syrian population has been living in poverty for the previous 13 years, which is associated with higher AF incidence.^5,11^ This inpatient cohort is relatively younger than patients admitted in high-income countries such as the US^12^ and neighbouring countries such as Jordan.^13^ This could be possibly due to the lack of primary or secondary care risk factor control owing to the lack of funding and resources. The high smoking rate likely affected our cohort’s AF incidence.^14^

Importantly, clinicians did appear to incorporate the latest evidence-based treatment guidelines to the best of their abilities,^15^ but limited resources are likely to have hindered this. Appropriate rate and rhythm control strategies were conducted using available resources, and DCCV was appropriately used in hemodynamically unstable patients. This contributed to the relatively acceptable inpatient outcomes, considering the challenging circumstances in Syria. There is ongoing evidence worldwide about the benefit of early rhythm control on symptoms, stroke risk, quality of life and CV outcomes, which can benefit this cohort.^16–18^ Expectedly, inpatient rhythm control was proven beneficial in SR reversion compared to. However, this approach could not be implemented due to the need for intensive follow-up, resources, and the unavailability of antiarrhythmic drugs and ablation centres in Syria.^19^ This may be an area for improvement to optimise long-term outcomes and avoid re-admissions, with follow-up studies recommended subsequently.

Regarding our thromboembolic events predictive model, it is unsurprising that a higher CHA2DS2-VASc score was associated with a higher thromboembolic risk in keeping with global data.^8^ However, thromboembolic events incidence of 11% in our cohort is relatively high compared to the risk for the corresponding CHA2DS2-VASc score (2.9%).^8^ Likely, these patients were not diagnosed in primary care, and anticoagulation was not started until acute presentation with a thromboembolic event or a complication of AF. This should push efforts through the Syrian health care system to utilise ECG in patients admitted to the emergency department to diagnose asymptomatic AF promptly.

Our model was highly predictive of inpatient CV and all-cause mortality. COPD was associated with increased mortality in previous studies.^20^ A history of thyroid disease is known to increase AF risk but has not been shown to increase mortality.^21^ Around 15% of our cohort with thyroid disease were on active treatment, which could have contributed to current results. To optimise AF treatment, active steps should be taken to educate patients about modifiable risk factors and lifestyle triggers. VHD is known to cause AF, and a community-based study suggested that moderate to severe biological VHD increases mortality.^22^ This can be reflected in our cohort, especially with the difficulty of accessing cardiac surgery services due to the poor/damaged infrastructure. Many of these patients remain untreated, which contributes to increased mortality. This highly predictive model can be used from a patient’s perspective to address smoking (which will improve COPD management) and encourage compliance with thyroid medications.

From a physician’s perspective, these models help identify patients at a high risk of deterioration to ensure early escalation and prompt treatment. The study is the first of its kind in Syria and would encourage more efforts to establish guidance to treat such vulnerable patients with limited resources. There remains a challenge to arrange long-term follow-ups after discharge to ensure SR maintenance in the form of clinic appointments, and Holter monitors in patients who were successfully treated as inpatients. On the other hand, in patients discharged in AF, there is a lack of a long-term follow-up to monitor left ventricular function, titrate medications, and discuss further treatment options, leading these patients to present in their extreme to the emergency department. This study opens the door for further AF-related research to assess outcomes in other cities. It encourages international organisations to support Syrian healthcare with infrastructures needed to optimise short- and long-term outcomes.

## Conclusion

Syrian inpatients admitted acutely with AF in Latakia are younger in comparison to neighbouring countries, and the limited resources prevented optimal AF management, especially elective rhythm control. The presence of thyroid disease, COPD and VHD were independent risk factors for CV and all-cause inpatient mortality. The risk of thromboembolic events increased with a higher CHA2DS2-VASc score.

## Limitations

Data collection was limited to a single tertiary care centre in Latakia. This city was relatively less affected by the Syrian conflict than the other northern and eastern regions of Syria. Therefore, our results might not be generalisable to other centres/regions, given the significant heterogeneity in the quality and level of hospital supplies and staffing. Additionally, our analysis included only routinely collected data within the medical records and by the number of patients who presented to the hospital. Therefore, other variables potentially impacting mortality may have yet to be identified.

## Supplemental Material


Supplemental Material - Atrial fibrillation inpatient management patterns and clinical outcomes during the conflict in Syria: An observational cohort study
Supplemental Material for Atrial fibrillation inpatient management patterns and clinical outcomes during the conflict in Syria: An observational cohort study by Ibrahim Antoun, Majed Aljabal Alkassem Alkhayer, Yaman Mahfoud, Alamer Alkhayer, Peter Simon, Ahmed Kotb, Joseph Barker, Akash Mavilakandy, Muhammad Usman Naseer, Riyaz Somani, G André Ng and Mustafa Zakkarin Perfusion.

## Data Availability

Data relating to this study are available upon reasonable request from the corresponding author.*

## References

[1] Rahman F Kwan GF Benjamin EJ . Global epidemiology of atrial fibrillation. Nat Rev Cardiol 2014; 11(11): 639. 25113750 10.1038/nrcardio.2014.118

[2] Al-Shamkhani W Ayetey H Lip GY . Atrial fibrillation in the Middle East: unmapped, underdiagnosed, undertreated. Expet Rev Cardiovasc Ther 2018; 16(5): 341–348. 10.1080/14779072.2018.1457953 29575965

[3] Akiki D El Hage S Wakim E , et al. Atrial fibrillation in the Arab world: a bibliometric analysis of research activity from 2004 to 2019. Journal of Cardiac Arrhythmias 2021; 34(1): 12–22.

[4] Alhaffar MB Janos S . Public health consequences after ten years of the Syrian crisis: a literature review. Glob Health 2021; 17: 1. 10.1186/s12992-021-00762-9 PMC8449996 34538248

[5] https://www.mercycorps.org/blog/facts-syria-crisis [accessed on 4/2/2024].

[6] Essien UR McCabe ME Kershaw KN , et al. Association between neighborhood-level poverty and incident atrial fibrillation: a retrospective cohort study. J Gen Intern Med 2022; 37(6): 1436–1443. 34240286 10.1007/s11606-021-06976-2 PMC9086074

[7] Vandenbroucke JP Von Elm E Altman DG , et al. Strengthening the reporting of observational studies in epidemiology (STROBE): explanation and elaboration. PLoS Med 2007; 4(10): e297. 17941715 10.1371/journal.pmed.0040297 PMC2020496

[8] Lip GY Nieuwlaat R Pisters R , et al. Refining clinical risk stratification for predicting stroke and thromboembolism in atrial fibrillation using a novel risk factor-based approach: the euro heart survey on atrial fibrillation. Chest 2010; 137(2): 263–272. 19762550 10.1378/chest.09-1584

[9] Thomas KL Jackson LR Shrader P , et al. Prevalence, characteristics, and outcomes of valvular heart disease in patients with atrial fibrillation: insights from the ORBIT‐AF (outcomes registry for better informed treatment for atrial fibrillation). J Am Heart Assoc 2017; 6(12): e006475. 29273635 10.1161/JAHA.117.006475 PMC5778999

[10] Lippi G Sanchis-Gomar F Cervellin G . Global epidemiology of atrial fibrillation: an increasing epidemic and public health challenge. Int J Stroke 2020; 16: 217. 31955707 10.1177/1747493019897870

[11] Essien UR McCabe ME Kershaw KN , et al. Association between neighborhood-level poverty and incident atrial fibrillation: a retrospective cohort study. Journal of general internal medicine 2022; 37: 1–8. special contribution of the European Heart Rhythm Association (EHRA) of the ESC. Eur Heart J. 2020; 42(5): 373–498. 10.1007/s11606-021-06976-2 PMC9086074 34240286

[12] Jackson SL Tong X Yin X , et al. Emergency department, hospital inpatient, and mortality burden of atrial fibrillation in the United States, 2006 to 2014. Am J Cardiol 2017; 120(11): 1966–1973. 28964382 10.1016/j.amjcard.2017.08.017 PMC6485413

[13] Hammoudeh A Khader Y Tabbalat R , et al. One-year clinical outcome in middle eastern patients with atrial fibrillation: the Jordan atrial fibrillation (JoFib) study. Int J Vasc Med 2022; 2022: 4240999. 35462945 10.1155/2022/4240999 PMC9020983

[14] Aune D Schlesinger S Norat T , et al. Tobacco smoking and the risk of atrial fibrillation: a systematic review and meta-analysis of prospective studies. Eur J Prev Cardiol 2018; 25(13): 1437–1451. 29996680 10.1177/2047487318780435

[15] Hindricks G Potpara T Dagres N , et al. 2020 ESC Guidelines for the diagnosis and management of atrial fibrillation developed in collaboration with the European Association for Cardio-Thoracic Surgery (EACTS) the Task Force for the diagnosis and management of atrial fibrillation of the European Society of Cardiology (ESC) Developed with the special contribution of the European Heart Rhythm Association (EHRA) of the ESC. Eur Heart J 2021; 42(5): 373–498. 32860505 10.1093/eurheartj/ehaa612

[16] Kirchhof P Camm AJ Goette A , et al. Early rhythm-control therapy in patients with atrial fibrillation. N Engl J Med 2020; 383(14): 1305–1316. 32865375 10.1056/NEJMoa2019422

[17] Guertin JR Dorais M Khairy P , et al. Atrial fibrillation: a real-life observational study in the Québec population. Can J Cardiol 2011; 27(6): 794–799. 21745721 10.1016/j.cjca.2011.03.009

[18] Kim D Yang P-S You SC , et al. Treatment timing and the effects of rhythm control strategy in patients with atrial fibrillation: nationwide cohort study. *BMJ*. 2021; 373: n991. 10.1136/bmj.n991 PMC8111568 33975876

[19] Fouad FM Sparrow A Tarakji A , et al. Health workers and the weaponisation of health care in Syria: a preliminary inquiry for the Lancet–American University of Beirut Commission on Syria. Lancet 2017; 390(10111): 2516–2526. 28314568 10.1016/S0140-6736(17)30741-9

[20] Ye J Yao P Shi X , et al. A systematic literature review and meta-analysis on the impact of COPD on atrial fibrillation patient outcome. Heart Lung 2022; 51: 67–74. 34740082 10.1016/j.hrtlng.2021.09.001

[21] Bruere H Fauchier L Brunet AB , et al. History of thyroid disorders in relation to clinical outcomes in atrial fibrillation. Am J Med 2015; 128(1): 30–37. 25058863 10.1016/j.amjmed.2014.07.014

[22] Benjamin EJ Levy D Vaziri SM , et al. Independent risk factors for atrial fibrillation in a population-based cohort. The Framingham Heart Study. JAMA 1994; 271(11): 840–4844. 8114238

